# Early-life determinants of cardiometabolic outcomes and accelerated biological ageing in Colombia

**DOI:** 10.18632/aging.206384

**Published:** 2026-05-26

**Authors:** Juan Carlos Rivillas, Emilie Courtin, Eleanor Winpenny, Oliver Robinson, Paolo Vineis

**Affiliations:** 1 Department of Epidemiology and Biostatistics, MRC Centre for Environment and Health, School of Public Health, Imperial College London, London, UK; 2 Department of Health Policy, London School of Economics and Political Sciences (LSE), London, UK; 3 School of Public Health, Imperial College London, London, UK; 4 Ageing and Epidemiology (AGE) Research Unit, School of Public Health, Imperial College London, London, UK

**Keywords:** adverse childhood experiences, forced childhood migration, biological ageing, cardiometabolic outcomes, life course epidemiology

## Abstract

Background: Adverse childhood experiences (ACEs) are critical early-life determinants of long-term health, yet their association with biological ageing and cardiometabolic risk remains poorly understood. We examined the association between ACEs, cardiometabolic outcomes and age acceleration among older adults in Colombia.

Methods: Data were drawn from 3,385 adults aged ≥60 years (1,726 women, 1,659 men) from the nationally representative Health, Well-Being, and Ageing Study (SABE-Colombia). Five ACEs before 15 years old were assessed: emotional abuse, domestic violence, poor self-reported health, scarcity of food, and forced childhood migration due to armed conflict. Biological ageing was estimated using Klemera-Doubal Method for Biological Age (∆KDMAge). Associations between ACEs and cardiometabolic outcomes (cardiovascular disease [CVD], diabetes, hypertension, and obesity) were evaluated using logistic and Poisson regression models; and associations with biological ageing using linear regression models, adjusting for sociodemographic factors.

Results: Among women, emotional abuse (OR=1.68), domestic violence (OR=1.55), scarcity of food (OR=1.44), and poor health status (OR=1.66) were associated with increased odds of CVD (OR=1.68). Among men, forced childhood migration was associated with higher risks of diabetes (OR=1.60), CVD (OR=1.55), and hypertension (OR=1.43). Forced childhood migration was also associated with age acceleration (∆KDMAge β =1.52), with a stronger association in women (∆KDMAge β =2.67). Dose-response associations were observed between cumulative ACEs and CVD in women and hypertension in men.

Conclusions: Early-life adversity, particularly forced childhood migration, is associated with higher cardiometabolic risk and accelerated biological ageing in later life, emphasizing the long-term biological costs of social and political instability.

## INTRODUCTION

Adverse Childhood Experiences (ACEs) have been linked to cardiometabolic outcomes and other health outcomes across the life course [1, 2]. Research has suggested an increased risk of cardiovascular disease [1, 3, 4], diabetes [5, 6], and obesity [5, 7, 8] among individuals exposed to one or more ACEs compared to non-exposed individuals. In previous studies exposure to emotional abuse or maltreatment during childhood doubled the likelihood of developing cardiometabolic diseases in adulthood [9–13]. Several meta-analyses have supported an association between single ACEs and health outcomes [7, 14, 15], and a dose-dependent relationship between cumulative ACEs and multimorbidity [16], violence, mental illness, and substance use [17]. In addition, exposures to ACEs are not equally distributed across genders. Prior research suggests that women are more likely to experience childhood adversity and have poorer health outcomes compared to men if they are exposed [1, 4]. However, one study in the UK found no gender differences [18].

The relationship between ACEs and later health outcomes can be understood through intermediate biological and psychosocial mechanisms. Chronic early-life stress may induce physiological dysregulation, including inflammation, hormonal imbalance, and accelerated biological aging [1, 19, 20]. These health mechanisms can be captured through markers of biological aging, which reflect the speed at which an individual’s biological age progresses relative to their chronological age [21, 22].

Intermediate biological mechanisms include stress-related dysregulation of neuroendocrine and inflammatory systems such as the hypothalamic–pituitary–adrenal (HPA) axis activation and chronic low-grade inflammation [23, 24]. People age at different rates due to a combination of genetic, environmental, and psychosocial factors, and understanding these differences remains a central challenge in longevity science. Integrating these mechanistic insights offers a theoretical framework to explain how early adversity “gets under the skin,” clarifying causal pathways and informing interventions aimed at mitigating long-term health risks [25]. A key objective in geroscience is to understand why and how we age, as biological age may better reflect an individual’s overall physiological state than chronological age [26, 27].

Life-course epidemiology demonstrates that early-life adversity is associated with long-term dysregulation of stress-response systems, including persistent activation of the HPA axis and elevated inflammatory burden [1, 19, 28]. HPA dysregulation links ACEs to accelerated aging through chronic cortisol elevation, increased allostatic load, and downstream metabolic disturbances [24]. ACE exposure is also associated with biomarkers of accelerated aging, including epigenetic age acceleration [14], shortened telomere length [29], and increased allostatic load [30, 31, 32]. Accelerated biological aging, in turn, is associated with higher risks of cardiometabolic disease, multimorbidity, functional decline, and mortality [21, 27, 32]. Although our cross-sectional study cannot formally test mediation pathways, these mechanisms provide conceptual framework for interpreting the observed associations.

Colombia is a particularly relevant country for the study of long-term consequence of ACEs, since a violent civil war occurred between 1964 and 2015 (Conflicto armado interno de Colombia, with more than 200,000 deaths [33]. We investigated the association between ACEs, including early forced migration related to the conflict, and cardiometabolic outcomes and biological age acceleration in a national sample of the Colombian population. The sample was recruited in 2015 and the average age was 69 years; therefore, a substantial proportion of participants had been exposed to the civil war during childhood and adolescence and a fraction of them migrated for this reason.

## RESULTS

### Study population and characteristics

Participants’ characteristics stratified by gender are presented in Table 1. In both samples (overall and biomarkers subsample), study participants were on average 69.1 (S.D. 7.05) years old and 56% were women. Most of the participants were Mestizo (42-44%), followed by White participants (27-29%). The study population had a high prevalence of low childhood socioeconomic position (73-79%), indicating an extensive experience of early life disadvantage within this generation.

**Table 1:** Baseline characteristics, ACEs, and cardiometabolic outcomes of the study participants by gender (SABE Colombia-2015 overall sample n= 18,044 and biomarker subsample n=3,385).

**Characteristics/ Categories**	**Overall sample**				**Subsample biomarkers**		
	**Total**	**Women**	**Men**		**Total**	**Women**	**Men**
N	18,044	10,101	7,943		3,385	2,026	1,359
**Sociodemographic characteristics**							
Mean chronological age, years^a^ (SD)	69.1 (7.05)	68.8 (6.98)	69.4 (7.13)		69.1 (7.07)	68.8 (6.94)	69.7 (7.24)
Mean biological age, years (SD)	NA	NA	NA		69.1 (9.77)	68.8 (9.34)	69.7 (10.4)
Ethnicity, n (%)							
Afro Colombian	2,125 (11.8%)	1,150 (11.4%)	975 (12.3%)		343 (10.13%)	195 (9.6%)	148 (10.8%)
Indigenous	1,438 (8.0%)	687 (6.8%)	751 (9.5%)		213 (6.2%)	89 (4.3%)	124 (9.1%)
Mestizo	7,757 (43.0%)	4,262 (42.2%)	3,495 (44.0%)		1432 (42.3%)	870 (42.9%)	562 (41.3%)
Other	1,772 (9.8%)	1,105 (9.9%)	667 (8.4%)		434 (12.8%)	282 (13.9%)	152 (11.1%)
White	4,952 (27.4%)	2,897 (28.7%)	2,055 (25.9%)		963 (28.4%)	590 (29.12%)	373 (27.4%)
Low childhood SEP (Yes)	10,728 (59.5%)	7,924 (78.4%)	6587 (82.9%)		2,032 (60%)	1,201 59.3%	831 (61.1%)
**Single adversity (age <15 years, Yes)**							
Emotional abuse, n (%)	792 (4.4%)	467 (4.6%)	325 (4.1%)		160 (4.7%)	104 (5.1%)	56 (4.1%)
Scarcity of food, n (%)	4,805 (26.6%)	2,537 (25.1%)	2,268 (28.6%)		898 (26.5%)	506 (25.0%)	392 (28.8%)
Poor childhood health, n (%)	2,168 (12.0%)	1,131 (11.2%)	1,037 (13.1%)		425 (12.6%)	253 (12.5%)	172 (12.7%)
Domestic violence, n (%)	2,984 (16.5%)	1,792 (17.7%)	1,192 (15.0%)		588 (17.4%)	372 (18.4%)	216 (15.9%)
Forced childhood migration^c^, n (%)	525 (2.9%)	291 (2.9%)	234 (2.9%)		99 (2.9%)	57 (2.8%)	43 (3.2%)
**Cumulative childhood adversity (age <15 years) ACEs score ^b^, n (%)**							
ACEs 0	5,156 (28.6%)	2,797 (27.7%)	2,359 (29.7%)		913 (27.0%)	532 (26.3%)	381 (28.0%)
ACEs 1	7,112 (39.4%)	4,051 (40.1%)	3,061 (38.5%)		1397 (41.3%)	847 (41.8%)	550 (40.5%)
ACEs 2	3,758 (20.8%)	2,141 (21.2%)	1,617 (20.4%)		554 (16.4%)	329 (16.2%)	225 (16.6%)
ACEs 3	1,551 (8.6%)	843 (8.3%)	708 (8.9%)		379 (11.2%)	232 (11.5%)	147 (10.8%)
ACEs 4+	467 (2.6%)	269 (2.7%)	198 (2.5%)		142 (4.2%)	86 (4.2%)	56 (4.3%)
**Adult cardiometabolic outcomes (age >60 years old), n (%)**							
Cardiovascular disease, n (%)	2,331 (12.9%)	1,355 (13.4%)	976 (12.3%)		458 (13.5%)	291 (14.4%)	167 (12.3%)
Diabetes, n (%)	2,925 (16.2%)	1,846 (18.3%)	1,079 (13.6%)		566 (16.7%)	369 (18.2%)	197 (14.5%)
Hypertension, n (%)	9,374 (52.0%)	5,818 (57.6%)	3,556 (44.8%)		1,847 (54.6%)	1,206 (59.5%)	641 (47.2%)
Obesity, n (%)	6,021 (33.4%)	3,877 (38.4%)	2,144 (27.0%)		862 (25.5%)	615 (30.4%)	247 (18.2%)

^a^Data is displayed as n (%) for categorical variables, mean (SD), and median for age. ^b^Cumulative ACEs score range 0-4 and more. ^c^Between 0-15 years old.

ACEs were also prevalent. Only 28.6% of the entire sample reported experiencing no ACEs, meaning over 70% had faced at least one significant adversity during childhood. The most commonly reported ACEs included scarcity of food (25-28%), poor childhood health (11-12%), and domestic violence (16-19%). A gender difference emerged in the experience of domestic violence, with women consistently reporting higher prevalence (19.2% overall sample; 18.4% in the biomarker subsample) compared to men (15% overall sample; 15.9% biomarker subsample). While other ACEs showed minor fluctuations between sexes, this particular difference was pronounced. The cumulative burden of ACEs also highlighted the widespread nature of these experiences, with a considerable proportion reporting multiple ACEs: two ACEs (16-20%), three ACEs (8-11%) and four or more ACEs (2-4%). Participants reporting more than one ACE were more likely to be women and non-white (p<0.001). Women more frequently reported emotional abuse and domestic violence compared to males. Men were more likely to report poor health status and scarcity of food. By ethnic groups, Indigenous and Afro-Colombian women had higher ACEs scores than White women and men.

### Cardiometabolic outcomes

Analysis of adult cardiometabolic outcomes showed significant gender differences. Hypertension was highly prevalent, affecting over half of the population (52%). Women showed a higher prevalence of hypertension (57.6% overall; 59.5% biomarker subsample) compared to men (52.6% overall; 59.6% biomarker subsample). Diabetes was also more prevalent among women than men (18% vs 13.6% overall sample). The most significant gender difference was observed in obesity, where women consistently showed a substantial higher prevalence (38.4% overall; 30.4% biomarker subsample) compared to men (27% overall; 18.2% biomarker subsample).

Supplementary Table 4 reports odds ratios and prevalence ratios for the association between ACEs and cardiometabolic outcomes in the overall sample. In adjusted models for demographic factors and low childhood SEP, women exposed to emotional abuse showed a strong and significant association with increased odds/prevalence of CVD (OR=1.68, PR=1.51, both *p*<0.001). This was not observed in men or for other cardiometabolic outcomes in either gender. Similar to emotional abuse, childhood maltreatment showed a strong and significant association with increased risk of CVD in women (OR=1.55, PR=1.44, both *p*<0.001), but this was not observed for other outcomes. Associations with CVD in men were less strong (OR=1.24, PR=1.20, p=0.020 and p=0.018 respectively).

Forced childhood migration was associated with increased risk of diabetes (OR=1.60, PR=1.47, p=0.005 and p=0.003 respectively), CVD (OR=1.55, PR=1.46, p=0.012 and p=0.006 respectively), and hypertension (OR=1.43, PR=1.20, p=0.008 and p=0.002 respectively) only among men. Scarcity of food was associated with increased risk of CVD in women (OR=1.44, PR=1.34, both *p*<0.001), but not with other outcomes. Poor childhood health was associated with increased risk of CVD (OR=1.66, PR=1.50, both *p*<0.001) in women and hypertension (OR=1.16, PR=1.09, p=0.027 and p=0.013) in men.

Supplementary Table 4 also illustrates the dose-response relationship between the cumulative adverse childhood experiences and the risk of cardiometabolic diseases, particularly strong among women. Tests for trend show that as the number of ACEs increases, the risks of cardiovascular diseases (*p*=<0.001), diabetes (women *p*=0.002 and men *p*=0.006) and hypertension (women *p*=0.005 and men *p*=0.0002) also increase. No significant dose-response association was observed between ACEs and obesity in either gender. In general, the confidence intervals for women were narrower than those for men.

### Biological age acceleration

We observed strong correlations between biological age and chronological age (KDMAge *r* = 0.83), which was expected since chronological age is a component of the KDM clock (Supplementary Table 3). Supplementary Table 5 reports the associations between adverse childhood experiences and biological ageing. Among all exposures, only forced childhood migration was associated with accelerated aging, with a stronger association in women. In the overall population, forced childhood migration was associated with 1.52-year higher biological age (∆KDMAge β =1.52, 95% CI = 0.57, 2.47, p=0.002). The association was substantially stronger in women (β =2.67, 95% CI = 1.24 – 4.10), indicating that women exposed to forced migration were on average, 2.67 years biologically older than their chronological age. Forced childhood migration was also associated with higher odds of accelerated ageing (OR=1.69, 95% CI = 1.09 – 2.48, p=0.019). These findings, suggest that exposures to early-life forced migration in conflict settings has a significant long-term impact on biological ageing, with greater vulnerability observed among women.

Poor childhood health was associated with higher biological age acceleration in later lifer (∆KDMAge β=0.53, 95% CI =0.04 – 1.01). In contrast, food scarcity during childhood showed a protective effect among men (β=-0.35, 95% CI= 0.73 – 0.03), suggesting lower biological age acceleration, although the association was not statistically significant. Similarly, cumulative childhood adversity in men was associated with lower biological age acceleration (β=-0.44; 95% CI -0.86 – 0.02). This unexpected finding may reflect unmeasured resilience pathways or suggesting a potential adaptative or selective-survivorship pattern or some type of bias; therefore, but this pattern requires cautious interpretation. No associations were observed between emotional abuse and accelerated biological aging. Adjusting for age, gender, ethnicity and low childhood SEP produced minimal changes in effects sizes or significant values, indicating that these factors were not major confounders.

## DISCUSSION

We found an association between adverse childhood experiences and the risk of CVD, diabetes, and hypertension in adulthood, with significant gender differences. Our findings are consistent with previous literature, in particular the studies by Zou et al. [10], Souama et al. [13] and Danese et al. [6] linking childhood adversity to elevated cardiometabolic risk. Specifically, we found that women exposed to emotional abuse, scarcity of food, and domestic violence during childhood had a substantially increased risk of CVD, ranging from 27%-71%.

We observed that men who migrated before age 15 had 55% higher odds of heart diseases and stroke, 60% higher odds of diabetes, and 43% higher odds of elevated blood pressure, while such increases were not found in women. These findings are consistent with previous research by Schooling et al. [34], who identified a sex-specific critical period in early childhood migration for the development of hypertension and heart disease in men (migration at ages 0-7 OR = 3.17, 95% CI: 1.70, 5.91).

We found a clear dose-response association between the number of ACEs and cardiovascular disease and diabetes risk, with women being more vulnerable (Supplementary Table 5). Women with three ACEs had a 148% higher risk of heart disease than those with minimal or no ACEs and were twice as likely to have higher odds of heart disease compared to males with similar ACEs exposure. These gender differences in dose-response are supported by existing literature [3, 35–37].

Overall, the higher odds of cardiovascular diseases among women with multiple ACEs may be explained by biological, socioeconomic and behavioural factors, and their cumulative consequences. Our findings suggest that specific ACEs in this population could potentiate each other when in combination and increase cardiovascular disease risk. Women are likely to experience heart disease in ways that are different from men [38, 39].

A key mechanistic insight that warrants emphasis is the role of neuroendocrine pathways [23, 24]. Chronic hypercortisolism and dysregulation of the hypothalamic–pituitary–adrenal axis represent critical mechanisms linking ACEs to accelerated biological aging. Persistent activation of the HPA axis can lead to increased allostatic load, chronic inflammation, and metabolic dysregulation, which collectively contribute to biological wear-and-tear and cardiometabolic risk [40, 41]. These pathways provide a plausible explanation for the observed associations between early-life adversity, sex-specific vulnerability, and age-related health outcomes.

The most important finding from our analyses is the novel connection between early forced childhood migration and accelerated biological ageing, but not with childhood emotional abuse, maltreatment, and scarcity of food; the latter observations contradict previous studies [42–46]. This is a relevant finding as previous studies linked childhood maltreatment to accelerated ageing [44–46] but not accelerated aging with forced childhood migration. Additionally, prior studies used prospective cohorts, and exposure to maltreatment was drawn from juvenile and adult court records rather than retrospective self-reported maltreatment like here, which is potentially subject to recall bias. Overall, across most ACEs types and biological ageing, we found a lack of statistically significant associations.

This study suggests that girls exposed to forced childhood migration during childhood tend to be biologically older than girls not exposed in later life. Previous research on the health impacts of forced childhood migration has primarily addressed women’s sexual, reproductive, and mental health [47, 48]. One study in the literature has reported that migration leads to the age acceleration of tissues within migrant groups compared to non-migrants [49]. The novel connection between this early life stressor and age acceleration in women needs to be studied using longitudinal data to elucidate underlying mechanisms linking these factors to accelerated ageing, particularly in the Colombian-specific context. Our findings are specific to a Colombian subsample of older individuals. The findings of our study (including internal migration and other ACEs) should be interpreted within the context of Colombia's civil war and terrorism that occurred when a large proportion of the SABE participants were children (1955 to 1965) and led to the death of 200,000 citizens. The pathways between ACEs and biological ageing may differ across populations, age groups, or types of ACEs.

Lastly, although dose-response relationships of cumulative ACEs with the acceleration of age in later life have been reported in previous studies [50–53] we did not observe such association in the SABE’s overall population. This finding is confirmed by a previous study [43] that used similar childhood adversity scores ranging from 0 to 3. The mixed evidence may be explained by the fact that studies used different operationalisations, types, and combinations of ACEs, and the use of broader ACE scores.

### Limitations of the study

There are several limitations that need to be considered. First, an important limitation is the use of a deterministic approach to model biological aging. Contemporary geroscience views aging as a non-linear, stochastic process. While the KDM provides mean estimates suitable for population-level analyses, it may not capture stochastics bursts of accelerated aging at the individual level. Future longitudinal studies may better capture these dynamics. Second, the study was based on a retrospective investigation of ACEs and other risk factors, i.e., the design is limited in its ability to establish causal associations and we may not have been able to account for all confounders. Third there was limited ACEs assessment in SABE. For example, there was no information on other vital ACEs such as physical and sexual child abuse, parental mental illness, unemployment, and racism. Fourth, SABE did not collect data on the context of exposure, duration, and severity. Fifth, self-reporting by older adults may introduce bias in ACEs recall or measurement. This could be particularly true for adversities like emotional abuse and domestic violence, which were still broadly socially accepted in the study context when adversities occurred in our population from 1955 to 1965. While people with self-reported diseases like cardiometabolic diseases can be biased in their recall of past ACEs, this is unlikely for people showing accelerated ageing based on biomarker measurements. A sixth concern is that the study relies on self-reported diagnoses for conditions such as hypertension and diabetes. While self-reports are commonly used in large epidemiological studies when clinical measurements are unavailable, they may result in underreporting of these conditions and could introduce bias.

A final concern is whether a customised version of the BA predictors can accurately compute the Klemera and Doubal index. Other studies have encountered this limitation due to data availability or the absence of biomarkers included in the original list. A number of studies using simplified biological ageing estimates, or different combinations of biomarkers to estimate BA suggests that these can be as reliable as traditional methods [46, 54–59]. Despite the extensive developments in quantifying BA rates, a universally accepted method for estimating BA is not currently available. In addition, studies in biological ageing show different methods of data collection, data availability, and association assumptions in different settings.

## CONCLUSIONS

Our study reinforced existing evidence from a Colombian population that adverse childhood experiences, particularly emotional abuse, domestic violence, food insecurity and poor early health elevate cardiovascular risk in older women, while forced childhood migration emerged as a driver of diabetes, hypertension in older men, and accelerated biological ageing in older women. Cumulative ACEs exposure was associated with increased CVD, hypertension and diabetes risk but not with markers of biological ageing, except in those who experienced forced childhood migration. These individuals exhibited signs of accelerated biological aging, which means they tend to be biologically older than adults not exposed to forced childhood migration, suggesting the enduring physiological imprint of early-life trauma linked to armed conflict. As one of the first studies to capture this association between forced childhood migration and age acceleration in a Latin American context, our findings suggest long-term biological costs of conflict related childhood adversity. Future research should build on this foundation with longitudinal data, broader population, and more diverse ACEs, particularly in LMICs.

## MATERIALS AND METHODS

### Study design and participants

We used data from the Health, Well-Being, and Ageing Study (SABE-Colombia, first wave), which employed multistage random cluster sampling across 240 municipalities. Of 36,153 eligible older adults, 23,694 were surveyed (66% response rate). The present study included 18,044 participants with complete ACEs and self-reported health data during household interviews (Supplementary Figure 1). A subsample of 4,092 individuals from the full cohort, randomly sampled from five major cities (Barranquilla, Bogotá, Cali, Medellín, and Bucaramanga) was selected for biomarker analysis (Supplementary Figure 2).

Each participant provided written informed consent, and a local ethics committee approved the study. Blood samples were collected using a vacutainer tube and stored at–80° C following standardized protocols. Further information on the representativeness of the sample, survey implementation, data collection, quality control, and ethical approval is provided elsewhere [60, 61]. The study excluded institutionalized older people or those with significant cognitive impairment. Data on social determinants, physical function, behaviors, self-reported health status, and blood samples were collected in 2015. Questionnaires were filled by interviewers, and blood pressure, anthropometric measurements and blood samples were taken at each respondent's residence.

### Adverse childhood experiences

We adapted a list of ACEs from World Health Organization (WHO) ACE-IQ questionnaire [62]. As the original SABE study did not capture all ACEs, we supplemented it with emotional abuse and forced migration available in other SABE’s questionnaires. Five ACEs were included: emotional abuse, scarcity of food, poor childhood health, domestic violence, and childhood migration. All responses were dichotomized (‘yes/one or more times’ vs. ‘no/never’) to indicate exposure. Forced childhood migration was defined as a positive response to having being ‘displaced due to armed conflict’ and based on reported date of forced migration prior to age 15.

### Biomarkers

We included 11 out of 17 markers representing cardiometabolic function for the quantification of individual biological ages, including triglycerides (mg/dL), glycated haemoglobin (HbA1c1) (%), low-density lipoprotein (LDL) cholesterol (mg/dL), high-density lipoprotein (HDL) cholesterol (mg/dL), non-HDL cholesterol (mg/dL), total cholesterol (mg/dL), systolic blood pressure (mmHg), arm circumference (cm), calf circumference (cm), body mass index (BMI) and A Body Shape Index (ABSI) [63]. ABSI is a measure of body shape that estimates risk of related abdominal obesity; while BMI only considers heigh and weight, ABSI also incorporates waist circumference to better account for the distribution of body fat, particularly visceral around the abdomen, which is a known risk factor for mortality [63]. The measurement protocols for these markers have been previously described [64, 65]. Diastolic blood pressure, pulse pressure, waist circumference, knee height, 30-Second Sit-to-Stand Test, and handgrip strength were excluded because they had missing values between 30%-49%. Height and weight were excluded to avoid multicollinearity with body mass index BMI and ABSI. Markers with skewed distributions were log-transformed (HbA1c1 and triglycerides).

### Biological age

The Klemera-Doubal Method for Biological Age (KDMAge) is reported in Supplementary Table 1 and was calculated using the *BioAge* R package [66, 67]. KDMAge predicts individuals’ biological age and provides insights into accelerated aging. KDMAge employs a relatively simple statistical model to predict BA based on a limited set of organ functions and inflammatory markers. KDMAge has been demonstrated to be a reliable predictor of mortality [68] and provides stable results for practical assessment of the BA [55]. In addition, as it provides a more accessible method for quantifying BA, it is one of the most cross-validated biological age indicators [52, 55, 56, 67, 69, 70].

For our linear regression models, we utilized ∆KDMAge residuals, where negative values indicate slower aging and positive values indicate faster aging. For our logistic regression, the continuous KDMAge variable was dichotomized to classify individuals into “KDMAge acceleration” or “KDMAge non-acceleration”. Participants with positive KDM Age values > chronological age were classified as “KDMAge acceleration”; while participants with negative or zero values < chronological age were classified as “KDMAge non-acceleration”. Supplementary Table 2 describes the models used for the KDMAge.

We validated KDM implementation by: i) replicating NHANES-derived parameters from Levine [71], This means that our implementation of KDMA biological age algorithm was correct by reproducing the same parameters previously derived from NHANES data; ii) confirming age-biomarker correlations in our data were within ranges consistent with prior KDM application (r=0.73-0.89), iii) verifying that the distribution of biological age and its correlations with chronological age were comparable to those reported in studies relative to prior studies using older populations [22, 53, 56, 72]; and iv) conducting a sensitivity analyses using alternative biomarkers combination separately for women, men, and the overall population. For each specification, KDM parameters were recalibrated and biological age acceleration recomputed; across models, the magnitude and direction of associations remained unchanged.

### Diseases in adulthood

SABE provides data on outcomes from self-reported diagnoses of cardiovascular diseases (CVD), hypertension (HTA), and diabetes. CVD includes self-reported episodes of heart attack, angina, and thrombosis. BMI was calculated based on physical measurements of height and weight (kg / [height (m)]^2^) and used a cut-off to indicate obesity (BMI >=30).

### Covariates

The covariates included in the analyses were demographic factors (age, sex, and ethnicity) and low childhood socioeconomic position. These variables were self-reported at wave 1, at the same time as the biological samples and measures were taken. The ethnic groups of the participants were Mestizo (mixed European and Amerindian heritage), white, Afro-Colombian (including raizales, palenqueros, and Afro-descended populations), indigenous, and others. Low childhood socioeconomic position was assessed using a question about socioeconomic position before 15 years old. Responses were categorized as high, intermediate, or low, and then dichotomised into “Yes” (low) and “No” (high or intermediate). Supplementary Figure 3 presents the Direct Acyclic Graph (DAG) illustrating the role of confounders and competing exposures in the relationship between ACEs, biological ageing, and health outcomes.

### Statistical analysis

To study ACE exposures, we first examined each ACE individually and then calculated an overall ACEs score [18] by summing the number of single adversities.

To examine the associations between single and cumulative ACEs with four diseases, we conducted logistic regression and Poisson regression analyses stratified by gender. We fitted two sets of models for each ACE operationalisation: a crude model and a model adjusted for age, ethnicity and low childhood SEP. To test the trend with the number of ACEs, we performed a Cochran Armitage test. Using both methods, odds ratios and prevalence ratios, provided a more complete understanding of the associations and ensured that results were not biased by method-specific limitations.

Associations between ACEs and biological age measures were estimated using (1) linear regression (continuous ∆KDMAge residual) and (2) logistic regression (dichotomous KDMAge acceleration as dependent variable). Results are interpretable as effect sizes on Beta coefficients and odds ratios, accompanied by 95% confidence intervals and p-values. A two-sided p-value of < 0.05 was considered statistically significant. Several sensitivity analyses were conducted by comparing results with published biological age estimates and by replicating the same models separately in the training and testing datasets.

All analyses were performed in R Studio version 4.1.2 (The R Foundation for Statistical Computing, Vienna, Austria). R packages used in the analysis included *compareGroups* to validate correlations, and the *ggbetweenstats* package [73] and the *Sjplot* package for regression modelling [74].

## Supplementary Material

Supplementary Figures

Supplementary Tables 1-3

Supplementary Table 4

Supplementary Table 5

## References

[1] Pitts C, Millar SR, Perry IJ, Phillips CM. Relationships between childhood adversity and inflammatory biomarkers in adulthood: A cross-sectional analysis of a middle-to older-aged population. SSM Popul Health. 2024; 25:101608. 10.1016/j.ssmph.2024.101608 38261965 PMC10797532

[2] Carroll JE, Gruenewald TL, Taylor SE, Janicki-Deverts D, Matthews KA, Seeman TE. Childhood abuse, parental warmth, and adult multisystem biological risk in the Coronary Artery Risk Development in Young Adults study. Proc Natl Acad Sci USA. 2013; 110:17149–53. 10.1073/pnas.1315458110 24062432 PMC3800991

[3] Zhang Z, Wang W, Yuan X, Wang X, Luo Y, Dou L, Zhang L, Wu M. Adverse childhood experiences and subsequent physical and mental health among young adults: Results from six universities in China. Psychiatry Res. 2024; 335:115832. 10.1016/j.psychres.2024.115832 38471243

[4] Qiao Y, Zhu D, Zhao M, Magnussen CG, Xi B. Adverse childhood experience, adopting a healthy lifestyle in adulthood, and risk of cardiovascular diseases. J Affect Disord. 2024; 362:450–8. 10.1016/j.jad.2024.07.023 39009308

[5] Wen S, Zhu J, Han X, Li Y, Liu H, Yang H, Hou C, Xu S, Wang J, Hu Y, Qu Y, Liu D, Aspelund T, et al. Childhood maltreatment and risk of endocrine diseases: an exploration of mediating pathways using sequential mediation analysis. BMC Med. 2024; 22:59. 10.1186/s12916-024-03271-9 38331807 PMC10854183

[6] Danese A, Moffitt TE, Harrington H, Milne BJ, Polanczyk G, Pariante CM, Poulton R, Caspi A. Adverse childhood experiences and adult risk factors for age-related disease: depression, inflammation, and clustering of metabolic risk markers. Arch Pediatr Adolesc Med. 2009; 163:1135–43. 10.1001/archpediatrics.2009.214 19996051 PMC3560401

[7] Wiss DA, Brewerton TD. Adverse Childhood Experiences and Adult Obesity: A Systematic Review of Plausible Mechanisms and Meta-Analysis of Cross-Sectional Studies. Physiol Behav. 2020; 223:112964. 10.1016/j.physbeh.2020.112964 32479804

[8] Waehrer GM, Miller TR, Silverio Marques SC, Oh DL, Burke Harris N. Disease burden of adverse childhood experiences across 14 states. PLoS One. 2020; 15:e0226134. 10.1371/journal.pone.0226134 31990910 PMC6986706

[9] Gilbert LK, Matthews S, Dube SR, Annor FB. Approaches for measuring cumulative childhood adversity: A study of youth from 5 sub-Saharan African countries. Child Abuse Negl. 2024; 150:106542. 10.1016/j.chiabu.2023.106542 37996356 PMC10961201

[10] Zou X, Zhao J, Feng A, Chan KH, Wu WC, Manson JE, Liu S, Li J. Adversities in childhood and young adulthood and incident cardiovascular diseases: a prospective cohort study. EClinicalMedicine. 2024; 69:102458. 10.1016/j.eclinm.2024.102458 38333371 PMC10850111

[11] Danese A, Tan M. Childhood maltreatment and obesity: systematic review and meta-analysis. Mol Psychiatry. 2014; 19:544–54. 10.1038/mp.2013.54 23689533

[12] Coelho R, Viola TW, Walss-Bass C, Brietzke E, Grassi-Oliveira R. Childhood maltreatment and inflammatory markers: a systematic review. Acta Psychiatr Scand. 2014; 129:180–92. 10.1111/acps.12217 24205846

[13] Souama C, Lamers F, Milaneschi Y, Vinkers CH, Defina S, Garvert L, Stein F, Woofenden T, Brosch K, Dannlowski U, Galenkamp H, de Graaf R, Jaddoe VW, et al., and EarlyCause consortium. Depression, cardiometabolic disease, and their co-occurrence after childhood maltreatment: an individual participant data meta-analysis including over 200,000 participants. BMC Med. 2023; 21:93. 10.1186/s12916-023-02769-y 36907864 PMC10010035

[14] Soares S, Rocha V, Kelly-Irving M, Stringhini S, Fraga S. Adverse Childhood Events and Health Biomarkers: A Systematic Review. Front Public Health. 2021; 9:649825. 10.3389/fpubh.2021.649825 34490175 PMC8417002

[15] Huang H, Yan P, Shan Z, Chen S, Li M, Luo C, Gao H, Hao L, Liu L. Adverse childhood experiences and risk of type 2 diabetes: A systematic review and meta-analysis. Metabolism. 2015; 64:1408–18. 10.1016/j.metabol.2015.08.019 26404480

[16] Senaratne DNS, Thakkar B, Smith BH, Hales TG, Marryat L, Colvin LA. The impact of adverse childhood experiences on multimorbidity: a systematic review and meta-analysis. BMC Med. 2024; 22:315. 10.1186/s12916-024-03505-w 39143489 PMC11325707

[17] Hughes K, Bellis MA, Hardcastle KA, Sethi D, Butchart A, Mikton C, Jones L, Dunne MP. The effect of multiple adverse childhood experiences on health: a systematic review and meta-analysis. Lancet Public Health. 2017; 2:e356–e366. 10.1016/S2468-2667(17)30118-4 29253477

[18] Lacey RE, Pinto Pereira SM, Li L, Danese A. Adverse childhood experiences and adult inflammation: Single adversity, cumulative risk and latent class approaches. Brain Behav Immun. 2020; 87:820–30. 10.1016/j.bbi.2020.03.017 32201253 PMC7327510

[19] Rivillas-García JC, Courtin E, Winpenny E, Adebayo-Clement O, Devia-Rodríguez R, Moreno-Mattar O, Vineis P. The intergenerational effects of low parental socio-economic position on cardiometabolic and inflammatory outcomes: A systematic review and meta-analysis. Eur J Clin Invest. 2026; 56:e70125. 10.1111/eci.70125 41020493 PMC12817245

[20] Joshi D, Gonzalez A, Lin D, Raina P. The association between adverse childhood experiences and epigenetic age acceleration in the Canadian longitudinal study on aging (CLSA). Aging Cell. 2023; 22:e13779. 10.1111/acel.13779 36650913 PMC9924940

[21] Belsky DW, Caspi A, Arseneault L, Baccarelli A, Corcoran DL, Gao X, Hannon E, Harrington HL, Rasmussen LJ, Houts R, Huffman K, Kraus WE, Kwon D, et al. Quantification of the pace of biological aging in humans through a blood test, the DunedinPoAm DNA methylation algorithm. Elife. 2020; 9:e54870. 10.7554/eLife.54870 32367804 PMC7282814

[22] Mak JK, McMurran CE, Kuja-Halkola R, Hall P, Czene K, Jylhävä J, Hägg S. Clinical biomarker-based biological aging and risk of cancer in the UK Biobank. Br J Cancer. 2023; 129:94–103. 10.1038/s41416-023-02288-w 37120669 PMC10307789

[23] Sluiskes MH, Goeman JJ, Beekman M, Slagboom PE, Putter H, Rodríguez-Girondo M. Clarifying the biological and statistical assumptions of cross-sectional biological age predictors: an elaborate illustration using synthetic and real data. BMC Med Res Methodol. 2024; 24:58. 10.1186/s12874-024-02181-x 38459475 PMC10921716

[24] Bell CG, Lowe R, Adams PD, Baccarelli AA, Beck S, Bell JT, Christensen BC, Gladyshev VN, Heijmans BT, Horvath S, Ideker T, Issa JJ, Kelsey KT, et al. DNA methylation aging clocks: challenges and recommendations. Genome Biol. 2019; 20:249. 10.1186/s13059-019-1824-y 31767039 PMC6876109

[25] Cao X, Yang G, Jin X, He L, Li X, Zheng Z, Liu Z, Wu C. A Machine Learning-Based Aging Measure Among Middle-Aged and Older Chinese Adults: The China Health and Retirement Longitudinal Study. Front Med (Lausanne). 2021; 8:698851. 10.3389/fmed.2021.698851 34926482 PMC8671693

[26] Belsky DW, Moffitt TE, Cohen AA, Corcoran DL, Levine ME, Prinz JA, Schaefer J, Sugden K, Williams B, Poulton R, Caspi A. Eleven Telomere, Epigenetic Clock, and Biomarker-Composite Quantifications of Biological Aging: Do They Measure the Same Thing? Am J Epidemiol. 2018; 187:1220–30. 10.1093/aje/kwx346 29149257 PMC6248475

[27] Levine ME, Lu AT, Quach A, Chen BH, Assimes TL, Bandinelli S, Hou L, Baccarelli AA, Stewart JD, Li Y, Whitsel EA, Wilson JG, Reiner AP, et al. An epigenetic biomarker of aging for lifespan and healthspan. Aging (Albany NY). 2018; 10:573–91. 10.18632/aging.101414 29676998 PMC5940111

[28] Danese A, McEwen BS. Adverse childhood experiences, allostasis, allostatic load, and age-related disease. Physiol Behav. 2012; 106:29–39. 10.1016/j.physbeh.2011.08.019 21888923

[29] Sumner JA, Colich NL, Uddin M, Armstrong D, McLaughlin KA. Early Experiences of Threat, but Not Deprivation, Are Associated With Accelerated Biological Aging in Children and Adolescents. Biol Psychiatry. 2019; 85:268–78. 10.1016/j.biopsych.2018.09.008 30391001 PMC6326868

[30] Misiak B, Stańczykiewicz B, Pawlak A, Szewczuk-Bogusławska M, Samochowiec J, Samochowiec A, Tyburski E, Juster RP. Adverse childhood experiences and low socioeconomic status with respect to allostatic load in adulthood: A systematic review. Psychoneuroendocrinology. 2022; 136:105602. 10.1016/j.psyneuen.2021.105602 34861465

[31] Finlay S, Roth C, Zimsen T, Bridson TL, Sarnyai Z, McDermott B. Adverse childhood experiences and allostatic load: A systematic review. Neurosci Biobehav Rev. 2022; 136:104605. 10.1016/j.neubiorev.2022.104605 35278597

[32] Jiang M, Tian S, Liu S, Wang Y, Guo X, Huang T, Lin X, Belsky DW, Baccarelli AA, Gao X. Accelerated biological aging elevates the risk of cardiometabolic multimorbidity and mortality. Nat Cardiovasc Res. 2024; 3:332–42. 10.1038/s44161-024-00438-8 39196113 PMC13265155

[33] Rivillas JC, Devia Rodriguez R, Song G, Martel A. How do we reach the girls and women who are the hardest to reach? Inequitable opportunities in reproductive and maternal health care services in armed conflict and forced displacement settings in Colombia. PLoS One. 2018; 13:e0188654. 10.1371/journal.pone.0188654 29346375 PMC5773007

[34] Schooling M, Leung GM, Janus ED, Ho SY, Hedley AJ, Lam TH. Childhood migration and cardiovascular risk. Int J Epidemiol. 2004; 33:1219–26. 10.1093/ije/dyh221 15567872

[35] Friedman EM, Karlamangla AS, Gruenewald TL, Koretz B, Seeman TE. Early life adversity and adult biological risk profiles. Psychosom Med. 2015; 77:176–85. 10.1097/PSY.0000000000000147 25650548 PMC4396808

[36] Jackson DB, Testa A, Woodward KP, Qureshi F, Ganson KT, Nagata JM. Adverse Childhood Experiences and Cardiovascular Risk among Young Adults: Findings from the 2019 Behavioral Risk Factor Surveillance System. Int J Environ Res Public Health. 2022; 19:11710. 10.3390/ijerph191811710 36141983 PMC9517189

[37] Miller NE, Lacey RE. Childhood adversity and cardiometabolic biomarkers in mid-adulthood in the 1958 British birth cohort. SSM Popul Health. 2022; 19:101260. 10.1016/j.ssmph.2022.101260 36238817 PMC9550530

[38] Johns Hopkins Medicine. Menopause and the Cardiovascular System [Internet]. 2024. Available from: https://www.hopkinsmedicine.org/health/conditions-and-diseases/menopause-and-the-cardiovascular-system

[39] Gillis EE, Sullivan JC. Sex Differences in Hypertension: Recent Advances. Hypertension. 2016; 68:1322–7. 10.1161/HYPERTENSIONAHA.116.06602 27777357 PMC5159215

[40] Pakulak E, Stevens C, Neville H. Neuro-, Cardio-, and Immunoplasticity: Effects of Early Adversity. Annu Rev Psychol. 2018; 69:131–56. 10.1146/annurev-psych-010416-044115 28846500

[41] Johnson SC, Cavallaro FL, Leon DA. A systematic review of allostatic load in relation to socioeconomic position: Poor fidelity and major inconsistencies in biomarkers employed. Soc Sci Med. 2017; 192:66–73. 10.1016/j.socscimed.2017.09.025 28963986

[42] Suglia SF, Clausing ES, Shelton RC, Conneely K, Prada-Ortega D, DeVivo I, Factor-Litvak P, Cirillo P, Baccarelli AA, Cohn B, Link BG. Cumulative Stress Across the Life Course and Biological Aging in Adulthood. Psychosom Med. 2024; 86:137–45. 10.1097/PSY.0000000000001284 38345302 PMC11001534

[43] Martinez RA, Howard AG, Fernández-Rhodes L, Maselko J, Pence BW, Dhingra R, Galea S, Uddin M, Wildman DE, Aiello AE. Does biological age mediate the relationship between childhood adversity and depression? Insights from the Detroit Neighborhood Health Study. Soc Sci Med. 2024; 340:116440. 10.1016/j.socscimed.2023.116440 38039767 PMC10843850

[44] Widom CS, Do HH, Miller QC, Javakhishvili M, Eckstein Indik C, Belsky DW. Childhood Maltreatment and Biological Aging in Middle Adulthood: The Role of Psychiatric Symptoms. Biol Psychiatry Glob Open Sci. 2024; 4:100341. 10.1016/j.bpsgos.2024.100341 39040430 PMC11260844

[45] Zannas AS. Biological aging and mental illness—a vicious cycle? JAMA Psychiatry. 2024; 81:433–4. 10.1001/jamapsychiatry.2024.0084 38477905

[46] Graf GH, Li X, Kwon D, Belsky DW, Widom CS. Biological aging in maltreated children followed up into middle adulthood. Psychoneuroendocrinology. 2022; 143:105848. 10.1016/j.psyneuen.2022.105848 35779342

[47] Rivillas JC, Devia-Rodriguez R, Ingabire MG. Measuring socioeconomic and health financing inequality in maternal mortality in Colombia: a mixed methods approach. Int J Equity Health. 2020; 19:98. 10.1186/s12939-020-01219-y 32731871 PMC7393844

[48] Calderón-Jaramillo M, Parra-Romero D, Forero-Martínez LJ, Royo M, Rivillas-García JC. Migrant women and sexual and gender-based violence at the Colombia-Venezuela border: a qualitative study. J Migr Health. 2020; 1–2:100003. 10.1016/j.jmh.2020.100003 34405159 PMC8352007

[49] Chilunga FP, Henneman P, Elliott HR, Cronjé HT, Walia GK, Meeks KA, Requena-Mendez A, Venema A, Bahendeka S, Danquah I, Adeyemo A, Klipstein-Grobusch K, Pieters M, et al. Epigenetic age acceleration in the emerging burden of cardiometabolic diseases among migrant and non-migrant African populations: the population based cross-sectional RODAM study. Lancet Healthy Longev. 2021; 2:E327–39. 10.1016/S2666-7568(21)00087-8 35146471 PMC7612337

[50] Bortz J, Guariglia A, Klaric L, Tang D, Ward P, Geer M, Chadeau-Hyam M, Vuckovic D, Joshi PK. Biological age estimation using circulating blood biomarkers. Commun Biol. 2023; 6:1089. 10.1038/s42003-023-05456-z 37884697 PMC10603148

[51] Kim K, Yaffe K, Rehkopf DH, Zheng Y, Nannini DR, Perak AM, et al. Association of adverse childhood experiences with accelerated epigenetic aging in midlife. JAMA Netw Open. 2023; 6:e2317987. 10.1001/jamanetworkopen.2023.17987 37306997 PMC10261996

[52] Gao X, Wang Y, Song Z, Jiang M, Huang T, Baccarelli AA. Early-life risk factors, accelerated biological aging and the late-life risk of mortality and morbidity. QJM. 2024; 117:257–68. 10.1093/qjmed/hcad247 37930885

[53] Mian O, Belsky DW, Cohen AA, Anderson LN, Gonzalez A, Ma J, Sloboda DM, Bowdish DME, Verschoor CP. Associations between exposure to adverse childhood experiences and biological aging: evidence from the Canadian Longitudinal Study on Aging. Psychoneuroendocrinology. 2022; 142:105821. 10.1016/j.psyneuen.2022.105821 35679774

[54] Murabito JM, Zhao Q, Larson MG, Rong J, Lin H, Benjamin EJ, Levy D, Lunetta KL. Measures of Biologic Age in a Community Sample Predict Mortality and Age-Related Disease: The Framingham Offspring Study. J Gerontol A Biol Sci Med Sci. 2018; 73:757–62. 10.1093/gerona/glx144 28977464 PMC5946832

[55] Jee H, Park J. Selection of an optimal set of biomarkers and comparative analyses of biological age estimation models in Korean females. Arch Gerontol Geriatr. 2017; 70:84–91. 10.1016/j.archger.2017.01.005 28110151

[56] Gaydosh L, Belsky DW, Glei DA, Goldman N. Testing Proposed Quantifications of Biological Aging in Taiwanese Older Adults. J Gerontol A Biol Sci Med Sci. 2020; 75:1680–5. 10.1093/gerona/glz223 31566204 PMC7494028

[57] Dalecka A, Bartoskova Polcrova A, Pikhart H, Bobak M, Ksinan AJ. Living in poverty and accelerated biological aging: evidence from population-representative sample of U.S. adults. BMC Public Health. 2024; 24:458. 10.1186/s12889-024-17960-w 38350911 PMC10865704

[58] Machado AV, Silva JFME, Colosimo EA, Needham BL, Maluf CB, Giatti L, Camelo LV, Barreto SM. Clinical biomarker-based biological age predicts deaths in Brazilian adults: the ELSA-Brasil study. Geroscience. 2024; 46:6115–26. 10.1007/s11357-024-01186-0 38753229 PMC11494676

[59] Chen H, Tang H, Zhang X, Huang J, Luo N, Guo Q, Wang X. Adherence to Life's Essential 8 is associated with delayed biological aging: a population-based cross-sectional study. Rev Esp Cardiol (Engl Ed). 2025; 78:37–46. 10.1016/j.rec.2024.04.004 38663840

[60] Ortega-Lenis D, Mendez F. Survey on health, well-being and aging. SABE Colombia 2015: Technical Report. Colomb Med (Cali). 2019; 50:128–38. 10.25100/cm.v50i2.4557 31607769 PMC6774577

[61] Gómez F, Osorio-García D, Panesso L, Curcio CL. Healthy aging determinants and disability among older adults: SABE Colombia. Rev Panam Salud Publica. 2021; 45:e98. 10.26633/RPSP.2021.98 34475887 PMC8369129

[62] World Health Organization (WHO). Adverse Childhood Experiences International Questionnaire (ACE-IQ). Available from: https://www.who.int/publications/m/item/adverse-childhood-experiences-international-questionnaire-(ace-iq)

[63] Bouchi R, Asakawa M, Ohara N, Nakano Y, Takeuchi T, Murakami M, Sasahara Y, Numasawa M, Minami I, Izumiyama H, Hashimoto K, Yoshimoto T, Ogawa Y. Indirect measure of visceral adiposity 'A Body Shape Index' (ABSI) is associated with arterial stiffness in patients with type 2 diabetes. BMJ Open Diabetes Res Care. 2016; 4:e000188. 10.1136/bmjdrc-2015-000188 27026809 PMC4800068

[64] Gomez F, Corchuelo J, Curcio CL, Calzada MT, Mendez F. SABE Colombia: Survey on Health, Well-Being, and Aging in Colombia-Study Design and Protocol. Curr Gerontol Geriatr Res. 2016; 2016:7910205. 10.1155/2016/7910205 27956896 PMC5124445

[65] Ministerio de Salud y Protección Social. Documento Metodológico Encuesta Nacional de Salud, Bienestar y Envejecimiento SABE Colombia. 2018; 27. Available from: https://www.minsalud.gov.co/sites/rid/Lists/BibliotecaDigital/RIDE/VS/ED/GCFI/doc-metodologia-sabe.pdf

[66] Kwon D, Belsky DW. A toolkit for quantification of biological age from blood chemistry and organ function test data: BioAge. Geroscience. 2021; 43:2795–808. 10.1007/s11357-021-00480-5 34725754 PMC8602613

[67] Mak JK, McMurran CE, Hägg S. Clinical biomarker-based biological ageing and future risk of neurological disorders in the UK Biobank. J Neurol Neurosurg Psychiatry. 2024; 95:481–4. 10.1136/jnnp-2023-331917 37926442 PMC11041565

[68] Buslova A. Prediction of Biological Age. 2017; 18. Available from: http://lup.lub.lu.se/student-papers/record/8917091

[69] Klemera P, Doubal S. A new approach to the concept and computation of biological age. Mech Ageing Dev. 2006; 127:240–8. 10.1016/j.mad.2005.10.004 16318865

[70] Chen L, Wu B, Mo L, Chen H, Zhao Y, Tan T, Chen L, Li Y, Yao P, Tang Y. Associations between biological ageing and the risk of, genetic susceptibility to, and life expectancy associated with rheumatoid arthritis: a secondary analysis of two observational studies. Lancet Healthy Longev. 2024; 5:e45–55. 10.1016/S2666-7568(23)00220-9 38081205

[71] Levine ME. Modeling the rate of senescence: can estimated biological age predict mortality more accurately than chronological age? J Gerontol A Biol Sci Med Sci. 2013; 68:667–74. 10.1093/gerona/gls233 23213031 PMC3660119

[72] Gao X, Geng T, Jiang M, Huang N, Zheng Y, Belsky DW, Huang T. Accelerated biological aging and risk of depression and anxiety: evidence from 424,299 UK Biobank participants. Nat Commun. 2023; 14:2277. 10.1038/s41467-023-38013-7 37080981 PMC10119095

[73] Patil I. Visualizations with statistical details: The ‘ggstatsplot’ approach. J Open Source Softw. 2021; 6:3167. Available from: https://joss.theoj.org/papers/10.21105/joss.03167

[74] Lüdecke D. Summary of Regression Models as HTML Table sjPlot. 2024. Available from: https://strengejacke.github.io/sjPlot/articles/tab_model_estimates.html

