# Supplementary material for: Early-life determinants of cardiometabolic outcomes and accelerated biological ageing in Colombia
Source: Aging (Albany NY). 2026 May 26;18(1):605–21. doi: 10.18632/aging.206384 (PMC13632847; doi:10.18632/aging.206384)
Supplement: Supplementary file 1 [file aging-18-1-206384-s001.pdf]

SUPPLEMENTARY FIGURES

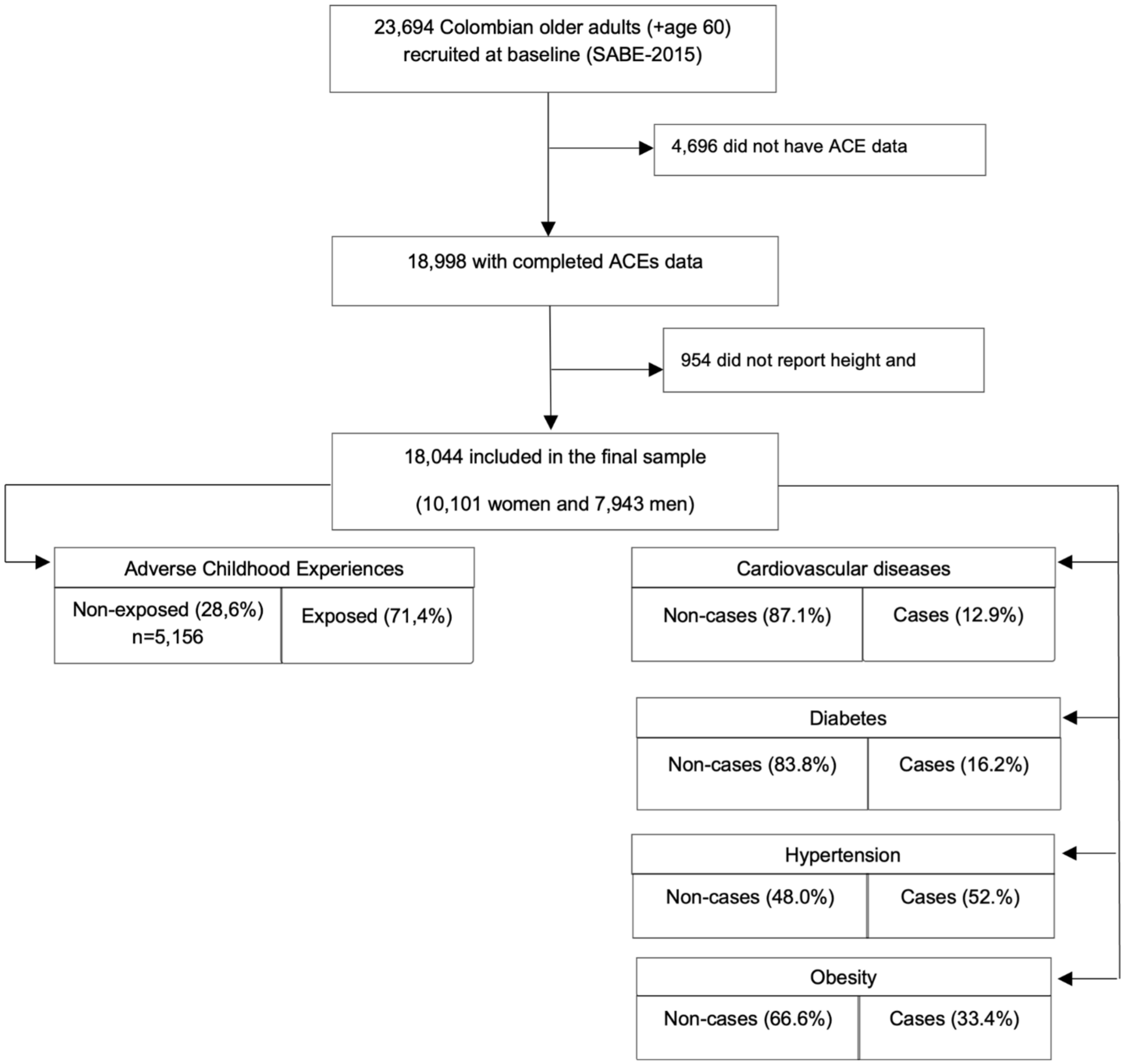

Supplementary Figure 1. Study population selection process and profile.

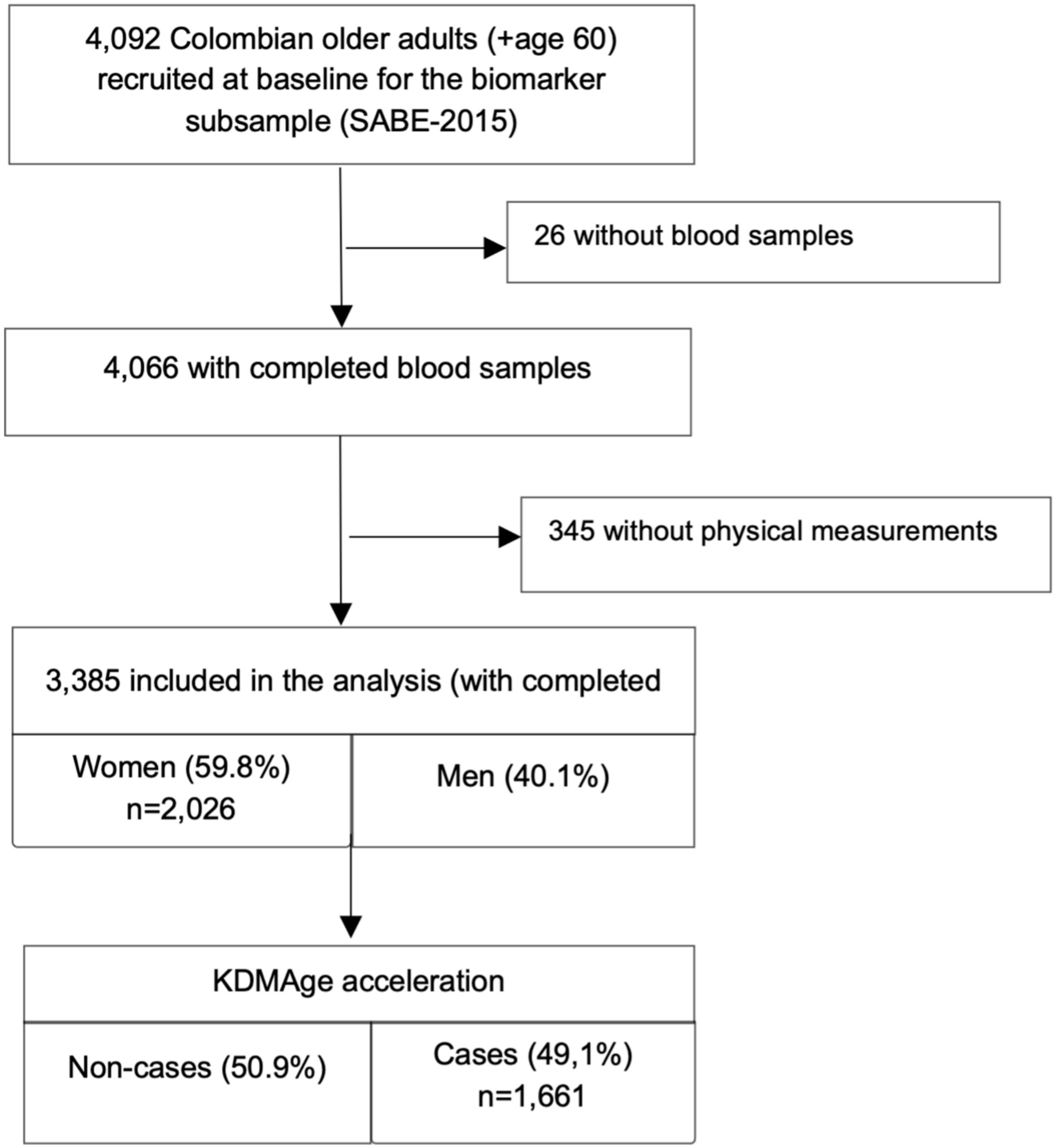

Supplementary Figure 2. Study population selection process and profile (Subsample of biomarkers).

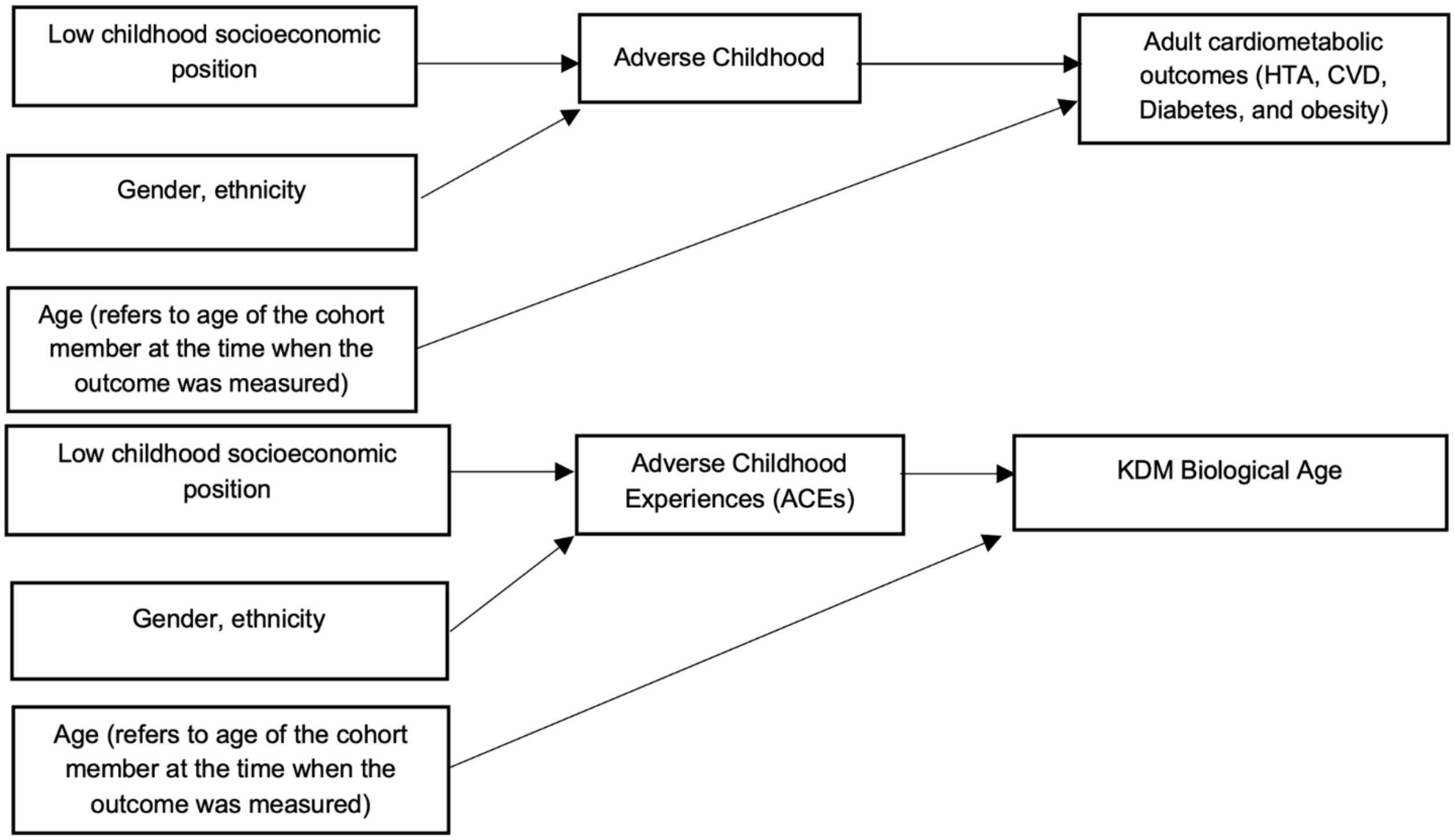

Supplementary Figure 3. This study is guided by the direct causal path and the indirect causal path of childhood adversity as exposures on adult health outcomes and biological ageing.
