# Supplementary material for: Early-life determinants of cardiometabolic outcomes and accelerated biological ageing in Colombia
Source: Aging (Albany NY). 2026 May 26;18(1):605–21. doi: 10.18632/aging.206384 (PMC13632847; doi:10.18632/aging.206384)
Supplement: Supplementary file 2 [file aging-18-1-206384-s002.pdf]

SUPPLEMENTARY TABLES

Please browse Full Text version to see the data of Supplementary Tables 4, 5.

Supplementary Table 1. Biomarkers Included in the SABE-COLOMBIA Biological Age Versions.

Biomarkers	KDMAge men	KDMAge women
Triglycerides	Yes	-
HbA1c1	Yes	Yes
Low-Density lipoprotein	Yes	Yes
High-Density Lipoprotein	Yes	-
Non-High-density Lipoprotein	Yes	Yes
Total cholesterol	Yes	Yes
Systolic blood pressure	Yes	Yes
A Body Shape Index	Yes	Yes
Body mass index	Yes	Yes
Calf circumference	Yes	Yes
Arm circumference	Yes	Yes

Supplementary Table 2. Definition and estimation of the Klemera-Doubal Method Biological Age (KDMAge).

Ítems	Klemera-Doubal Method Biological Age (KDMAge)
Description	KDMAge is computed based on regression models of biomarkers on age and represents the predicted physiological age of an individual (72).
Methods	The equation takes information from m number of regression lines of chronological age regressed on m biomarkers: $KDM\ Biological\ Age = \frac{\sum_{j=1}^{m}\left(x_j - q_j\right)\frac{k_j}{S_j^2} + \frac{a}{S_{BA}^2}}{\sum_{j=1}^{m}\left(\frac{k_j}{S_j}\right)^2 + \frac{1}{S_{BA}^2}}$
Interpretation	Positive KDMAge residual suggests that the individual is aging faster than their chronological age, meaning their biological age is higher than their actual age. ΔKDMAge residual values greater than 0, suggest faster biological ageing, whereas values less than or equal to 0, indicates slower biological ageing.
Advantages	Provides reliable and stable results and has been validated in different population samples.

Supplementary Table 3. Summary statistics for the study population with biomarkers available.

Characteristics	Women (N=2,026)	Men (N=1,359)	Overall (N=3,385)
Chronological age (year), mean ± SD[a]	68.8 (6.94)	69.7 (7.24)	69.1 (7.07)
Biological age KDMAge (years), mean ± SD[a]	68.8 (9.34)	69.7 (10.4)	69.1 (9.77)
ΔKDMAge residual	0.00 (6.25)	0.00 (7.42)	0.00 (6.74)
KDMAge acceleration [b]	1,006 (49.7%)	655 (48.2%)	1,661 (49.1%)
KDMAge non-acceleration	1,020 (50.3%)	704 (51.8%)	1,724 (50.9%)
**Biomarkers included in KDMAge algorithms, mean ±SD**			
HbA1c1 (%)	13.4 (1.28)	14.1 (1.23)	13.6 (1.31)
Low-density lipoprotein LDL (mg/dL)	127 (31.8)	120 (31.5)	124 (31.9)
High-density lipoprotein HDL (mg/dL)	46.5 (11.1)	41.2 (10.6)	44.4 (11.2)
Total Cholesterol (mg/dL)	200 (38.0)	185 (37.4)	194 (38.4)
Non-HDL (mg/dL)	154 (38.2)	144 (37.3)	150 (38.1)
Triglycerides (mg/dL)	151 (53.9)	144 (54.0)	148 (54.0)
Glucose (mg/dL)	93.9 (9.30)	93.4 (9.76)	93.7 (9.49)
SBP (mm Hg)	138 (23.9)	140 (25.0)	139 (24.3)
Calf circumference (cm)	34.8 (3.85	34.5 (3.42)	34.7 (3.68)
Arm circumference (cm)	29.2 (4.14)	28.0 (3.25)	28.7 (3.85)
A body shape index (ABSI)	0.082 (0.006)	0.083 (0.005)	0.083 (0.006)
Body-mass index BMI	27.8 (4.99)	26.3 (4.18)	27.2 (4.74)

(a) Included 3,385 participants for analysis. Mean values (standard deviation) for continuous variables and n (%) for categorical variables. (b) Employed to construct KDMAge. (c) Cardiovascular diseases, diabetes and hypertension self-reported. (e) BMI ≥ 30 kg/m² or higher defines obesity.
