# Supplementary material for: Early-life determinants of cardiometabolic outcomes and accelerated biological ageing in Colombia
Source: Aging (Albany NY). 2026 May 26;18(1):605–21. doi: 10.18632/aging.206384 (PMC13632847; doi:10.18632/aging.206384)
Supplement: Supplementary file 3 [file aging-18-1-206384-s003.docx]

Supplementary Table 4. Analysis of Associations Between Adverse Childhood Experiences (ACEs) and Cardiometabolic Outcomes in Adulthood, Stratified by Gender in Older Adults in Colombia (using overall sample n=18,044).

| **ACEs type/**  **Outcomes** | **Women** | | | | | | | | **Men** | | | | | | | |
| --- | --- | --- | --- | --- | --- | --- | --- | --- | --- | --- | --- | --- | --- | --- | --- | --- |
|  | **CVD** | | **Diabetes** | | **Hypertension** | | **Obesity** | | **CVD** | | **Diabetes** | | **Hypertension** | | **Obesity** | |
|  | **Coefficients (95% CI)** | ***P-* value** | **Coefficients (95% CI)** | ***P-* value** | **Coefficients (95% CI)** | ***P-* value** | **Coefficients (95% CI)** | ***P-* value** | **Coefficients (95% CI)** | ***P-* value** | **Coefficients (95% CI)** | ***P-* value** | **Coefficients (95% CI)** | ***P-* value** | **Coefficients (95% CI)** | ***P-* value** |
| **Emotional abuse** (Reference=non-exposed) | | | | | | | | | | | | | | | | |
| Odds Ratios | 1.68  (1.32-2.12) | **<0.001** | 1.09 (0.86-1.37) | 0.479 | 1.20  (0.98-1.45) | 0.069 | 1.18 (0.98-1.43) | 0.086 | 1.32 (0.95-1.80) | 0.090 | 0.98 (0.69-1.35) | 0.903 | 1.21  (0.96-1.52) | 0.103 | 1.20 (0.94-1.52) | 0.145 |
| Prevalence Ratios | 1.51  (1.20-1.87) | **0.0002** | 1.08  (0.86-1.34) | 0.401 | 1.07  (0.94-1.20) | 0.077 | 1.11  (0.95-1.28) | 0.055 | 1.23  (0.90-1.64) | 0.164 | 1.00  (0.72-1.35) | 0.956 | 1.08  (0.91-1.27) | 0.166 | 1.13  (0.91-1.38) | 0.160 |
| **Childhood maltreatment** (Reference=non-exposed) | | | | | | | | | | | | | | | | |
| Odds Ratios | 1.55  (1.35-1.79) | **<0.001** | 1.11  (0.97-1.30) | 0.111 | 1.03 (0.93-1.15) | 0.538 | 0.96 (0.86-1.07) | 0.474 | 1.24 (1.03-1.49) | **0.020** | 1.13 (0.94-1.34) | 0.183 | 1.05 (0.92-1.19) | 0.456 | 1.10 (0.96-1.26) | 0.174 |
| Prevalence Ratios | 1.44  (1.26-1.64) | **<0.001** | 1.07  (0.94-1.20) | 0.201 | 1.01  (0.94-1.08) | 0.505 | 0.99  (0.91-1.08) | 0.898 | 1.20  (1.01-1.43) | **0.018** | 1.02  (0.93-1.12) | 0.100 | 1.02  (0.93-1.12) | 0.410 | 1.07  0.95-1.20 | 0.149 |
| **Forced childhood migration** (Reference=non-exposed) | | | | | | | | | | | | | | | | |
| Odds Ratios | 1.00  (0.70-1.38) | 0.981 | 1.05 (0.77-1.40) | 0.768 | 0.96 (0.75-1.22) | 0.716 | 1.11 (0.87-1.41) | 0.389 | 1.55 (1.09-2.15) | **0.012** | 1.60 (1.14-2.20) | **0.005** | 1.43 (1.10-1.86) | **0.008** | 1.21 (0.91-1.60) | 0.180 |
| Prevalence Ratios | 0.98  (0.69-1.33) | 0.897 | 1.05  (0.79- 1.36) | 0.662 | 0.97  (0.83-1.14) | 0.685 | 1.05  (0.86-1.26) | 0.477 | 1.46  (1.06-1.97) | **0.006** | 1.47  (1.08-1.96) | **0.003** | 1.20  (0.99- 1.42) | **0.002** | 1.15  0.90-1.45 | 0.140 |
| **Scarcity of food** (Reference=non-exposed) | | | | | | | | | | | | | | | | |
| Odds Ratios | 1.44  (1.26-1.64) | **<0.001** | 1.12 (1.00-1.27) | 0.056 | 1.06 (0.96-1.16) | 0.258 | 0.90 (0.82-0.99) | **0.032** | 1.19 (1.02-1.38) | **0.026** | 1.01 (0.87-1.16) | 0.933 | 1.10 (0.99-1.22) | 0.066 | 0.89 (0.80-1.00) | 0.053 |
| Prevalence Ratios | 1.34  (1.18-1.52) | **<0.001** | 1.07  (0.96- 1.19) | 0.149 | 1.01  (0.95-1.08) | 0.391 | 0.94  (0.86-1.01) | **0.049** | 1.13  (0.98-1.30) | 0.058 | 1.00  (0.87- 1.15) | 0.902 | 1.06  0.98- 1.14 | **0.034** | 0.82-1.01 | **0.045** |
| **Childhood poor health status** (Reference=non-exposed) | | | | | | | | | | | | | | | | |
| Odds Ratios | 1.66  (1.41-1.94) | **<0.001** | 1.09  (0.93-1.27) | 0.259 | 0.99 (0.87-1.13) | 0.901 | 0.97 (0.85-1.10) | 0.631 | 1.15 (0.94-1.39) | 0.160 | 1.20 (1.00-1.44) | 0.051 | 1.16  (1.03-1.35) | **0.027** | 0.93 (0.80-1.07) | 0.278 |
| Prevalence Ratios | 1.50  (1.29-1.74) | **<0.001** | 1.07  (0.92- 1.23) | 0.272 | 0.98  (0.90-1.07) | 0.710 | 0.96  (0.87-1.07) | 0.450 | 1.12  (0.93-1.35) | 0.163 | 1.19  (1.00-1.41) | 0.022 | 1.09  (0.98-1.20) | **0.013** | 0.93  (0.82-1.06) | 0.278 |
| **Cumulative ACEs** (Reference=ACEs 0) | | | | | | | | | | | | | | | | |
| ACEs 1 | | | | | | | | | | | | | | | | |
| Odds Ratios | 1.32  (1.13-1.54) | **0.001** | 1.04  (0.91-1.18) | 0.590 | 1.11  (1.01-1.23) | **0.034** | 0.88  (0.79-0.97) | **0.009** | 1.10  (0.93-1.31) | 0.256 | 0.98  (0.83-1.15) | 0.769 | 1.16  (1.03-1.29) | **0.010** | 0.86  (0.76-0.97) | **0.012** |
| Prevalence Ratios | 1.26  (1.09-1.47) | **0.0009** | 1.02  (0.91- 1.15) | 0.662 | 1.05  (0.98-1.12) | **0.019** | 0.93  (0.86-1.01) | **0.028** | 1.10  (0.94-1.29) | 0.182 | 0.98  (0.84-1.15) | 0.860 | 1.08  (0.99-1.17) | **0.013** | 0.88  (0.79- 0.97) | **0.005** |
| ACEs 2 | | | | | | | | | | | | | | | | |
| Odds Ratios | 1.77  (1.49-2.10) | **<0.001** | 1.11  (0.96-1.29) | 0.166 | 1.20  (1.06-1.35) | **0.003** | 0.85  (0.76-0.96) | **0.009** | 1.22  (1.00-1.48) | 0.051 | 1.11  (0.92-1.34) | 0.258 | 1.24  (1.09-1.42) | **<0.001** | 0.82  (0.71-0.95) | **0.009** |
| Prevalence Ratios | 1.59  (1.35-1.88) | **<0.001** | 1.07  (0.93- 1.23) | 0.233 | 1.07  (0.99-1.16) | **0.004** | 0.92  (0.83-1.01) | **0.027** | 1.16  (0.96-1.39) | 0.094 | 1.09  (0.91-1.30) | 0.270 | 1.12  (1.02-1.24) | **0.0009** | 0.86  (0.76- 0.98) | **0.010** |
| ACEs 3 | | | | | | | | | | | | | | | | |
| Odds Ratios | 2.47  (1.99-3.05) | **<0.001** | 1.27  (1.04-1.54) | **0.018** | 1.17  (0.99-1.37) | 0.058 | 0.97  (0.83-1.14) | 0.695 | 1.32  (1.02-1.69) | **0.032** | 1.29  (1.01-1.63) | **0.036** | 1.25  (1.05-1.48) | **0.013** | 0.91  (0.75-1.10) | 0.333 |
| Prevalence Ratios | 2.09  (1.71-2.54) | **<0.001** | 1.23  (1.02- 1.46) | **0.009** | 1.06  (0.95-1.18) | 0.064 | 0.99  (0.87-1.12) | 0.961 | 1.28  (1.01-1.62) | **0.024** | 1.26  (1.01-1.56) | **0.021** | 1.13  (0.99-1.28) | **0.008** | 0.91  (0.77-1.07) | 0.208 |
| ACEs 4+ | | | | | | | | | | | | | | | | |
| Odds Ratios | 2.45  (1.75-3.38) | **<0.001** | 1.40  (1.03-1.89) | **0.030** | 1.28  (0.92-1.55) | 0.082 | 0.79  (0.61-0.82) | 0.082 | 1.40  (0.90-2.11) | 0.117 | 1.36  (0.90-1.99) | 0.131 | 1.62  (1.21-2.18) | **0.001** | 0.94  (0.67-1.29) | 0.690 |
| Prevalence Ratios | 2.07  (1.51-2.78) | **<0.001** | 1.27  (0.96- 1.67) | **0.048** | 1.07  (0.90-1.26) | 0.177 | 0.87  (0.70-1.07) | 0.112 | 1.35  (0.89-1.97) | 0.096 | 1.31  (0.90-1.86) | 0.101 | 1.28  (1.04-1.57) | **0.0004** | 0.94  (0.70-1.23) | 0.619 |

Notes for interpretation: (a) CI confidence interval, Model adjusted for demographic factors (Age, gender, and ethnicity); and childhood socioeconomic position. Odds Ratios (ORs), Prevalence Ratios (PRs).
