# Supplementary material for: Early-life determinants of cardiometabolic outcomes and accelerated biological ageing in Colombia
Source: Aging (Albany NY). 2026 May 26;18(1):605–21. doi: 10.18632/aging.206384 (PMC13632847; doi:10.18632/aging.206384)
Supplement: Supplementary file 4 [file aging-18-1-206384-s004.docx]

Supplementary Table 5. Associations of adverse childhood experiences with biological age acceleration (using the biomarker subsample n=3,385).

| Predictors/outcomes | **Overall older population** | | | | **Man** | | | | **Woman** | | | |
| --- | --- | --- | --- | --- | --- | --- | --- | --- | --- | --- | --- | --- |
|  | **Continuous**  **∆KDMAge residual** | | **Categorical KDM**  **BA acceleration** | | **Continuous**  **∆KDMAge residual** | | **Categorical KDM**  **BA acceleration** | | **Continuous**  **∆KDMAge residual** | | **Categorical KDM**  **BA acceleration** | |
|  | **Β 95 % CI** | **P-value** | **Odds ratio 95% CI** | **P-value** | **Β 95 % CI** | **P-value** | **Odds ratio 95% CI** | **P-value** | **Β 95 % CI** | **P-value** | **Odds ratio 95% CI** | **P-value** |
| **Emotional abuse** (Reference=non-exposed) | | | | |  |  |  |  |  |  |  |  |
| Unadjusted model | 0.14  (-0.61 – 0.89) | 0.713 | 0.86 (0.63 – 1.19) | 0.372 | -0.90 (-1.85 – 0.06) | 0.065 | 0.45  (0.25-0.80) | 0.008 | 0.71 (-0.35 – 1.77) | 0.191 | 1.19  (0.80-1.78) | 0.381 |
| Multivariate model^c^ | 0.12  (-0.64 – 0.87) | 0.765 | 0.87 (0.63 – 1.19) | 0.382 | -0.85 (-1.81 – 0.10) | 0.080 | 0.45  (0.25-0.80) | 0.008 | 0.64 (-0.43 – 1.71) | 0.241 | 1.16  (0.78-1.74) | 0.463 |
| **Childhood maltreatment** (Reference=non-exposed) | | | | |  |  |  |  |  |  |  |  |
| Unadjusted model | -0.30  (-0.72 – 0.12) | 0.159 | 0.89 (0.74 – 1.06) | 0.188 | -0.74 (-1.26 – -0.22) | **0.005** | 0.68  (0.51-0.92) | 0.011 | -0.04 (-0.65 – 0.56) | 0.891 | 1.01  (0.81-1.27) | 0.922 |
| Multivariate model^c^ | -0.33  (-0.75 – 0.10) | 0.137 | 0.89 (0.74 – 1.07) | 0.201 | -0.67 (-1.19 – -0.15) | **0.012** | 0.71 (0.52-0.95) | 0.023 | -0.10 (-0.71 – 0.52) | 0.757 | 1.03  (0.82-1.29) | 0.793 |
| **Forced childhood migration** (Reference=non-exposed) | | | | |  |  |  |  |  |  |  |  |
| Unadjusted model | 1.51 (0.56 – 2.45) | **0.002** | 1.62 (1.08 – 2.45) | **0.021** | -0.05 (-1.13 – 1.04) | 0.929 | 0.69  (0.37-1.28)_ | 0.250 | 2.70 (1.27 – 4.12) | **<0.001** | 3.46  (1.90-6.73) | **<0.001** |
| Multivariate model^c^ | 1.52 (0.57 – 2.47) | **0.002** | 1.64 (1.09 – 2.48) | **0.019** | 0.02 (-1.07 – 1.10) | 0.977 | 0.70  (0.37-1.30) | 0.268 | 2.67 (1.24 – 4.10) | **<0.001** | 3.46  (1.90-6.74) | **<0.001** |
| **Scarcity of food** (Reference=non-exposed) | | | | |  |  |  |  |  |  |  |  |
| Unadjusted model | -0.32  (-0.68 – 0.04) | 0.082 | 0.87 (0.75 – 1.02) | 0.078 | -0.41 (-0.83 – 0.01) | 0.057 | 0.78  (0.62-0.99) | 0.043 | -0.26 (-0.80 – 0.28) | 0.348 | 0.95  (0.77-1.16) | 0.590 |
| Multivariate model^c^ | -0.35  (-0.73 – 0.03) | 0.068 | 0.88 (0.75 – 1.03) | 0.110 | -0.23 (-0.67 – 0.20) | 0.291 | 0.84  (0.66-1.08) | 0.176 | -0.40 (-0.97 – 0.16) | 0.164 | 0.91  (0.74-1.13) | 0.390 |
| **Childhood poor health status** (Reference=non-exposed) | | | | |  |  |  |  |  |  |  |  |
| Unadjusted model | 0.52  (0.04 – 1.00) | **0.035** | 1.08 (0.88 – 1.33) | 0.439 | 0.26 (-0.31 – 0.83) | 0.373 | 1.06  (0.77-1.46) | 0.732 | 0.69 (-0.02 – 1.40) | 0.056 | 1.10  (0.85-1.44) | 0.470 |
| Multivariate model^c^ | 0.53  (0.04 – 1.01) | **0.032** | 1.09 (0.89 – 1.34) | 0.405 | 0.27 (-0.30 – 0.84) | 0.352 | 1.07  (0.77-1.47) | 0.702 | 0.70 (-0.01 – 1.41) | 0.054 | 1.10  (0.85-1.44) | 0.471 |
| **Cumulative ACEs** (reference: Zero ACEs) | | | | |  |  |  |  |  |  |  |  |
| Unadjusted model | -0.34  (-0.69 – 0.02) | 0.067 | 0.92  (0.79-1.08) | 0.314 | -0.48 (- 0.90 - -0.06) | **0.025** | 0.82  (0.65-1.04) | 0.106 | -0.23 (-0.77 - -0.30) | 0.389 | 1.00  (0.82-1.22) | 0.987 |
| Multivariate model^c^ | -0.33  (-0.68 – 0.03) | 0.076 | 0.93  (0.80-1.08) | 0.327 | -0.44 (-0.86 – 0.02) | **0.039** | 0.84  (0.66-1.06) | 0.144 | -0.21 (-0.74 - -0.33) | 0.446 | 1.01  (0.83-1.23) | 0.933 |

Notes for interpretation: CI confidence interval, Models: (a) unadjusted models; and (b) Multivariate model (Age, gender, ethnicity, and childhood socioeconomic position-adjusted model). Beta coefficient should be interpreted in years in KDMAge.
